# Emergy-Based Sustainability Evaluation and Optimization Scenarios for 341 Chinese Home-Cooked Dishes

**DOI:** 10.3390/foods15183204

**Published:** 2026-09-10

**Authors:** Yanhui Guo, Wansong Zong, Wen Zhang

**Affiliations:** School of Geography and Environment, Shandong Normal University, Jinan 250358, China; 2023010120@stu.sdnu.edu.cn

**Keywords:** emergy analysis, input structure, sustainable food systems, resource efficiency, regional cuisines, scenario analysis, food processing, home-cooked dishes

## Abstract

Chinese home-cooked dishes (CHCDs) link agricultural production and daily consumption, but their resource dependence and sustainability remain poorly quantified. We standardized 341 recipes from China’s 34 provincial-level administrative regions, conducted a production-to-consumption analysis using emergy (solar-equivalent available energy directly and indirectly required for a product or service), and evaluated six optimization scenarios. Mean emergy input was 5.93 × 10^12^ sej·dish^−1^, with nonrenewable purchased inputs accounting for 77.99%. Ingredients, oil and sauces, and cooking contributed 69.17%, 21.69%, and 9.14%, respectively. Plant-based production relied on labor and chemical inputs; animal-based production relied on feed. Ruminant meats had relatively high nonrenewable inputs and environmental loads; vegetables had lower values. Across CHCDs, the emergy sustainability index (ESI), transformity, emergy consumption intensity, and food processing intensity averaged 0.26, 1.02 × 10^6^ sej·J^−1^, 1.45 × 10^12^ sej·serving^−1^, and 4.64 × 10^11^ sej·serving^−1^, respectively. Sustainability declined from plant-based through mixed to animal-based dishes. Shandong cuisine ranked highest and Zhejiang cuisine lowest in combined sustainability and efficiency. Combining crop–livestock recycling, mechanization and energy-efficiency improvements, technological advancement and scaling up, moderate oil and sauce reduction, and cooking-efficiency improvements was projected to increase ESI by 19.34% relative to baseline. These results support dish-level emergy accounting for improving agri-food sustainability.

## 1. Introduction

Global agri-food systems are under growing pressure to meet rising food demand while reducing resource depletion and environmental degradation [1]. A meta-analysis of global projections estimated that food demand could increase by 35–56% between 2010 and 2050 [2]. Concurrently, agri-food systems account for roughly 33% of global greenhouse gas emissions and 80% of total freshwater withdrawals [3,4], exacerbating the strain on global ecological boundaries. These environmental burdens extend far beyond farm-gate production, as post-farm operations such as processing, distribution, and culinary preparation also entail substantial additional resource demands that compound supply-chain complexity [5]. Because consumers choose and combine foods as dishes rather than as isolated commodities, dish-level analysis can reveal how ingredient composition and preparation shape resource use and environmental impacts [6]. It can therefore connect upstream production processes with practical consumption-side interventions.

Chinese home-cooked dishes (CHCDs) are a major component of everyday diets in China and encompass diverse combinations of plant- and animal-based ingredients, condiments, and cooking methods, offering an intuitive reflection of China’s agricultural production structure and dietary characteristics [7]. Environmental assessments have begun to quantify this diversity. Yang et al. evaluated greenhouse gas emissions from 540 dishes across 36 cuisines and estimated that China’s food system emitted approximately 4.64 Gt CO_2_-eq in 2020, equivalent to 37% of national emissions [8]. Zheng et al. compared carbon, water, and ecological footprints across eight culinary traditions and identified pronounced differences among regional cuisines [9]. These studies establish the value of dish-level assessment but generally apply aggregated environmental coefficients to ingredients. Consequently, variation in upstream agricultural practices and inputs—such as machinery, labor, feed, fertilizers, pesticides, energy, and fixed assets—remains insufficiently resolved. This limits the ability to determine how farm-stage resource dependence propagates to dish-level sustainability and where coordinated interventions would be most effective.

Emergy represents the cumulative available energy consumed directly and indirectly in the transformations required to produce a product or deliver a service [10]. Emergy analysis provides a complementary systems framework for addressing this gap by converting heterogeneous inputs to a common solar-energy-equivalent basis, expressed in solar emjoules (sej) [11]. By bridging agricultural production and household consumption, this methodology clarifies the interactions among resource inputs, energy transformations, and environmental pressures across the dish-level production-to-consumption chain [12]. Unlike conventional environmental footprint and material flow approaches, emergy accounting evaluates inputs on the basis of the cumulative energy required for their formation, thereby providing a common metric across production and consumption stages [13]. Crucially, it traces upstream agricultural processes to quantify resource inputs, energy transformations, and system efficiency, including contributions from natural resources, agrochemicals, fossil energy, labor, and fixed assets [14,15]. Recent applications indicate that emergy-based scenarios, such as crop–livestock recycling and energy-efficiency improvements, can reduce reliance on nonrenewable resources while improving overall system sustainability [16,17]. Consequently, emergy analysis provides an analytical tool for mapping dish-level resource structures, identifying critical resource-use hotspots, and evaluating optimization scenarios across agri-food supply chains [12,18].

Accordingly, this study applies emergy analysis to 341 selected CHCD recipes covering China’s 34 provincial-level administrative regions and diverse culinary categories. It addresses three questions: (1) How do emergy input structures and sustainability indicators vary among dishes, dish types, and regional cuisines? (2) Which ingredients, production inputs, and life-cycle stages dominate nonrenewable resource dependence and environmental loading? (3) How much can sustainability improve under individual and combined optimization scenarios? This study provides a new perspective for understanding the resource and environmental implications of China’s regional dietary systems and offers a scientific basis for promoting the transition of agri-food systems toward greater resource efficiency and environmental sustainability.

## 2. Materials and Methods

### 2.1. Study Scope and Sample

We analyzed a collection of 341 selected CHCD recipes covering China’s 34 provincial-level administrative regions. The sample encompassed seven broad dish categories—cold dishes, soups, meat dishes, soybean products, vegetable dishes, flour-based staple foods, and desserts—as well as eleven primary cooking techniques, including stir-frying, steaming, braising, and deep-frying. CHCDs incorporate diverse products from both the crop and livestock sectors and reflect recipe-level ingredient combinations and culinary practices under contemporary consumption regimes [19]. Furthermore, because the standardized recipe database provides a consistent basis for comparing dishes across regions, CHCDs effectively link upstream agricultural resource allocation with downstream culinary preparation, thereby enabling a high-resolution evaluation of dietary-energy delivery at the final-consumption stage.

We categorized the CHCDs into three distinct types: plant-based dishes (PBD, *n* = 101), animal-based dishes (ABD, *n* = 110), and mixed plant–animal dishes (MPAD, *n* = 130) (Table A1 presents 20 representative examples). The primary ingredients were derived from 24 benchmark agricultural commodities: 6 animal-based products (pork, beef, mutton, chicken, egg, and milk) and 18 plant-based products (tangerine, peanut, soybean, paddy rice, apple, wheat, maize, pepper, kidney bean, mandarin orange, cucumber, eggplant, tomato, cauliflower, cabbage, radish, potato, and Chinese cabbage). Through primary processing, these 24 commodities yielded 188 secondary ingredients, including rice, tofu, and cured meat. Because the corresponding upstream production systems span diverse agroecological regions across China, they provide a baseline for characterizing the environmental attributes, sustainability, and optimization potential of the CHCD system. Notably, aquatic products (e.g., fish, shrimp, and shellfish) were excluded from the assessment framework because of data constraints. Current national and provincial panel datasets provide only coarse aquaculture statistics, such as yield and fry counts, and lack detailed inventories of key upstream inputs, including feed, labor, energy, disinfectants, and fixed assets. Consequently, aquaculture could not be incorporated into a unified emergy accounting framework alongside crop, livestock, and poultry production.

### 2.2. Recipe Collection and Standardization

#### 2.2.1. Standardization of Ingredient Data for CHCDs

Before evaluating the agricultural inputs, nutritional energy content, and environmental pressures associated with individual dishes, we systematically constructed a CHCD recipe database. Recipes were obtained from a classic culinary publication [20] and the home-cooking section of a leading culinary platform [21]. A total of 341 recipes were selected based primarily on the availability of standardized recipes with sufficiently complete information for consistent dish-level emergy accounting. The required information included ingredient composition and quantities, cooking oil and sauce inputs, cooking methods and durations, recommended serving sizes, and regional origin. The number of dishes originating from each of the 34 provincial-level administrative regions is reported in Table A2. Ingredient names were subsequently harmonized, and quantities were standardized as follows: (1) the quantities of all oils and sauces were converted using the measurement units specified in the recipes (1 tablespoon = 15 mL; 1 teaspoon = 5 mL); (2) ambiguous descriptions of condiment quantities were converted into specific values based on Wei et al. [22]; (3) the weights of individual vegetables were standardized using data from the U.S. Department of Agriculture [23] (Table A3); and (4) cooking durations corresponding to low, medium, and high heat settings were classified and quantified to ensure accurate estimates of natural gas and electricity consumption (Table A4). This rigorous standardization provided a reliable basis for the subsequent energy accounting and emergy analysis.

#### 2.2.2. Energy Standardization of CHCDs

The household dietary energy provided by each CHCD recipe at the consumption stage was quantified based on ingredient quantities, with corrections for edible fractions and the application of energy-content values obtained primarily from the Chinese Food Composition Database [24]. During cooking, edible oils and sauces are rarely consumed in full; some adhere to cookware or tableware, whereas others are discarded with residual broth. The unconsumed proportion varies substantially among cooking methods; for example, floating oil in high-oil dishes is rarely consumed in its entirety. Consequently, treating raw recipe inputs as equivalent to actual intake would systematically overestimate dietary energy. To address this issue, we introduced an attachment coefficient, the oil absorption rate (OAR), analogous to the post-cooking retention factors used in food composition research, to convert raw input quantities into the amounts actually ingested. Here, the OAR represents the proportion of the input oil and sauce that is ultimately consumed. For dishes exhibiting features of multiple cooking categories, a strict classification hierarchy was established to ensure mutually exclusive categorization, with priority assigned in the following descending order: deep-frying, high-oil dishes, red braising, soups, stir-frying, and mixing. Specifically, the OAR values were set at 72% for stir-frying, 8% for deep-frying, 55% for high-oil dishes, 92% for mixing, 88% for red braising, and 95% for soups [25,26].

The edible mass of each CHCD was calculated as follows:

(1) $$W=\sum_{j=1}^{m}\left(W_{ing,j}\times k_{ing,j}\right)+\;\left(W_{oil}+\;W_{sauce}\right)\times OAR$$

where *W* denotes the total edible mass of a dish; *m* denotes the number of ingredients included in the summation; $W_{ing,j}$ and $k_{ing,j}$ denote the raw mass and edible fraction of the *j*th ingredient, respectively; $W_{oil}$ and $W_{sauce}$ denote the recipe-specified input amounts of edible oil and sauce, respectively; and OAR denotes the oil absorption rate.

The total dietary energy provided by a dish at the household consumption stage was calculated as follows:

(2) $$Q=\sum_{i=1}^{n}\left(\frac{W_{i}}{100}\times N_{i}\right)$$

where *Q* denotes the total dietary energy provided by a dish (kcal); *n* denotes the total number of components included in the summation; *i* indexes the constituent ingredients, oil, and sauce; $W_{i}$ denotes the corrected edible mass of component *i* (g); and $N_{i}$ denotes the corresponding energy content (kcal/100 g). To enable transformity-based accounting within the emergy framework, all energy values were converted from kilocalories to joules (1 kcal = 4184 J).

### 2.3. Emergy Synthesis

#### 2.3.1. System Boundary Definition

**Figure 1:**
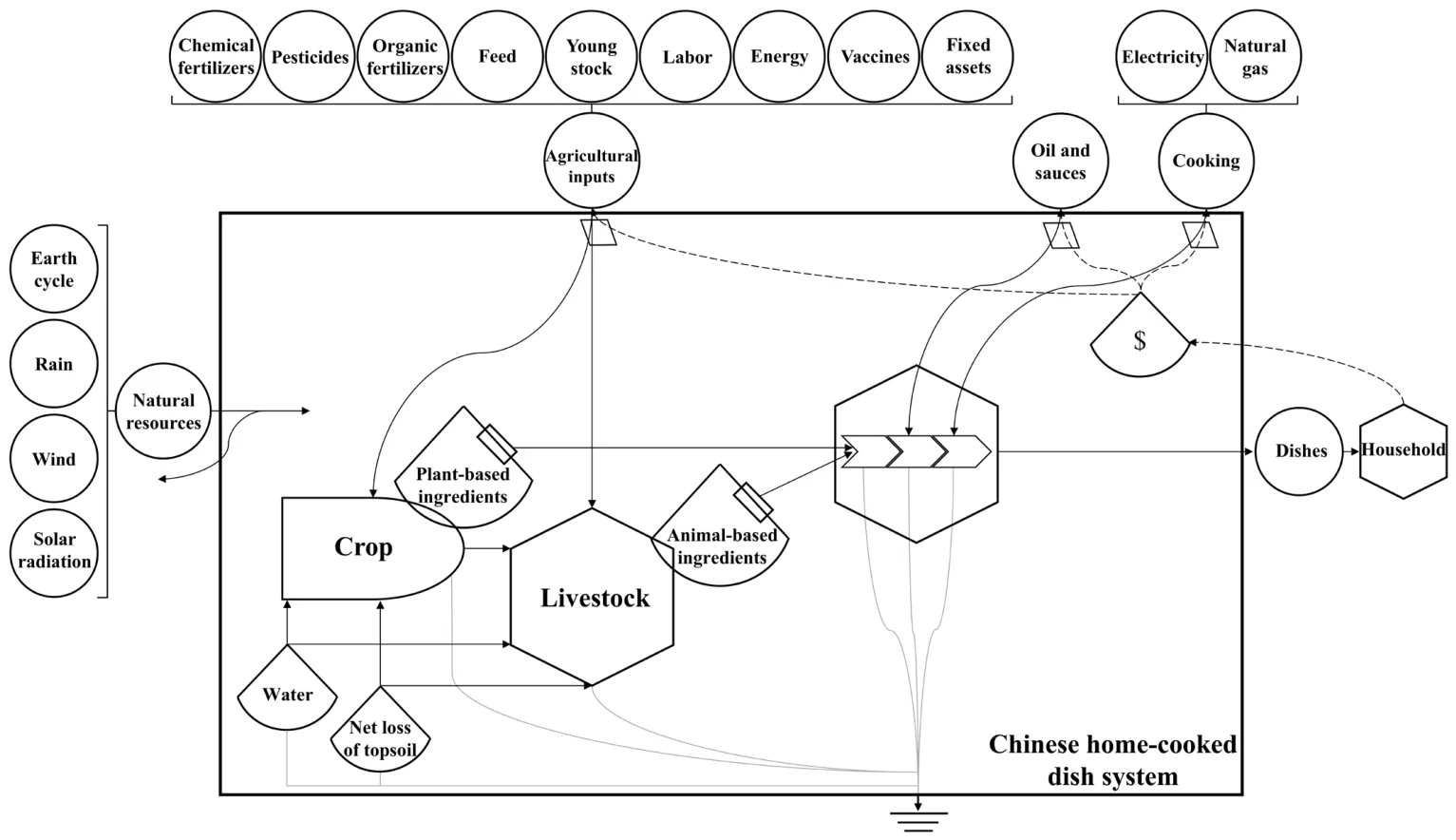
Schematic emergy flow diagram of the CHCD system.

We first defined the temporal and process boundaries of the CHCD system in both temporal and spatial dimensions. Temporally, Chinese agricultural production statistics for 2024 were used as the baseline for characterizing the upstream supply of ingredients. In terms of process scope, the system boundary encompassed the production of raw ingredients, the processing of oils and sauces, and the preparation of final dishes. An emergy flow diagram was constructed using standard emergy symbols [27] to illustrate the system structure and internal dynamics (Figure 1). System inputs comprised local natural resources (e.g., solar radiation, precipitation, water, and soil) and purchased inputs (e.g., agrochemicals, feed, energy, labor, and fixed assets), whereas system outputs comprised final dishes and the energy embodied in them. Solid and dashed arrows represent emergy and monetary flows, respectively, thereby illustrating the major relationships among processes and providing a theoretical basis for evaluating resource interactions and overall system sustainability.

#### 2.3.2. Emergy Accounting and Unit Emergy Values

Energy (J), mass (g), and monetary flows (USD) were converted to a common unit of solar emjoules (sej) by multiplying each flow by its corresponding unit emergy value (UEV), thereby enabling all resources within the production system to be evaluated on a consistent basis. The equations used to calculate the emergy of environmental and purchased resources are presented in Table 1. In accordance with the criteria for UEV selection commonly adopted in emergy analyses of agricultural systems [28] (see Appendix B for details), the UEVs used in this study were obtained from authoritative sources in the emergy literature (Table 2) and rescaled to the global emergy baseline of 12 × 10^24^ sej·yr^−1^ [29]. Specifically, because no published UEVs were directly applicable to cooking oils or sauces, the UEVs for these two inputs were estimated by accounting for both the primary production of oil crops and sugar crops and the energy consumed during food processing (Appendix B.1 and Appendix B.2). Based on the total emergy inputs thus accounted for, a renewability factor was introduced for each system input to distinguish between the renewable and nonrenewable components utilized and embodied in its production, thereby enhancing the scientific rigor of the system evaluation. Accordingly, the total emergy input of each CHCD was calculated as follows:

(3) $$U=\sum_{j=1}^{m}(E_{\mathrm{ing},j}\times k_{raw,j})+E_{\mathrm{oil}}+E_{\mathrm{sauces}}+E_{\mathrm{cooking}}$$

where U denotes the total emergy input of a dish; $E_{\mathrm{ing},j}$ denotes the emergy input associated with the production system for the *j*th ingredient; $k_{raw,j}$ denotes the conversion coefficient used to express the *j*th ingredient in terms of its primary agricultural commodity equivalent; and $E_{\mathrm{oil}}$, $E_{\mathrm{sauces}}$, and $E_{\mathrm{cooking}}$ denote the emergy inputs associated with cooking oil, sauces, and culinary preparation, respectively.

(4) $$R+FR=\sum_{i=1}^{n}\left(E_{i}\times RNF_{i}\right)$$

(5) N+FN=U−R−FR

where *R* denotes renewable natural emergy; *FR* denotes the renewable component of purchased emergy; $E_{i}$ denotes the *i*th emergy input; $RNF_{i}$ denotes the renewability factor representing the renewable fraction of the *i*th emergy input; N denotes nonrenewable natural emergy; *FN* denotes the nonrenewable component of purchased emergy; and *U* denotes the total emergy input of a dish.

#### 2.3.3. Emergy-Based Sustainability Indices

We first employed conventional emergy indices [42] to evaluate the production performance, environmental pressure, and sustainability of the CHCD system. The emergy yield ratio (EYR) measures the system’s capacity to utilize local resources and contribute to the broader economy; a higher EYR indicates greater resource-use capacity and a larger contribution to the broader economy. The environmental loading ratio (ELR) quantifies the environmental pressure imposed by the production activities of the system; a higher ELR indicates a greater environmental burden. The emergy sustainability index (ESI) characterizes system sustainability as the ratio of EYR to ELR; a higher ESI indicates greater system sustainability. Transformity (TRA) was calculated as the total emergy input required per unit of dietary energy supplied; a lower TRA indicates that less emergy is required to supply one unit of dietary energy and thus reflects higher production efficiency. These indices were calculated as follows:

(6) $$EYR=\frac{U}{FN+FR}$$

(7) $$ELR=\frac{N+FN}{R+FR}$$

(8) $$ESI=\frac{EYR}{ELR}$$

(9) $$TRA=\frac{U}{Q}$$

where *U* is the total emergy input per dish; *FN* and *FR* denote the nonrenewable and renewable components of purchased emergy, respectively; *N* and *R* denote the nonrenewable and renewable components of local natural emergy, respectively; and *Q* is the total dietary energy supplied by a dish.

Based on the emergy evaluation framework recommended by Lan et al. [43], we developed two new consumption-oriented indicators to assess the sustainability of the CHCD system across the production and consumption stages. Emergy consumption intensity (ECI) quantifies the total emergy requirement per recipe serving; a higher ECI indicates greater emergy demand to provide one serving. Food processing intensity (FPI) quantifies the emergy inputs associated with cooking oils, sauces, and cooking energy per recipe serving. A higher FPI indicates greater preparation-related emergy demand per serving.

(10) $$ECI=\frac{U}{P}$$

(11) $$FPI\;=\frac{E_{\mathrm{oil}}+E_{\mathrm{sauces}}+E_{\mathrm{cooking}}}{P}$$

where *U* is the total emergy input per dish; *P* is the recommended number of servings for each dish, as specified in the corresponding recipe; and $E_{\mathrm{oil}}$, $E_{\mathrm{sauces}}$, and $E_{\mathrm{cooking}}$ denote the emergy inputs associated with cooking oils, sauces, and cooking energy, respectively.

### 2.4. Design of Optimization Scenarios for the CHCD System

Based on the main intervention domains represented within the CHCD system boundary—upstream agricultural inputs, cooking oil and sauce use, and cooking energy consumption (Figure 1)—we established a business-as-usual baseline and six optimization scenarios to explore sustainable development pathways for the CHCD system (Table 3). The business-as-usual (BAU) scenario served as the reference scenario, under which all baseline parameters used in the emergy accounting were kept unchanged, including ingredient composition and quantities, agricultural production inputs, oil and sauces, cooking methods and energy consumption, and the corresponding unit emergy values. No optimization measures were introduced under the BAU scenario, and its results were used as the benchmark for evaluating the six optimization scenarios. Under the integrated crop–livestock recycling (ICLR) scenario, a crop–livestock recycling strategy was implemented in upstream agricultural production to enhance resource cascading and circularity within the system. Digestate was applied as fertilizer in feed-crop cultivation, while biogas produced through the anaerobic digestion of livestock manure was used to generate electricity. Under the enhanced mechanization and energy efficiency (EMEE) scenario, the level of mechanization in upstream agricultural production was increased, and machinery operation was optimized to improve energy-use efficiency and reduce fossil-fuel inputs. Under the technological advancement and appropriate scaling-up (TAAS) scenario, advanced farming technologies adopted from Australia and North America were introduced into upstream agricultural production to improve fertilizer- and feed-use efficiency, enable precision pesticide application, promote the use of low-opportunity-cost feeds, and facilitate the transition of smallholder farms to appropriately scaled operations, thereby strengthening agrochemical input management. Under the moderate oil and sauce reduction (MOSR) scenario, cooking oil and sauce inputs for each dish were uniformly reduced by 10% and 20%, respectively, relative to their corresponding BAU values. This scenario was designed to evaluate how reductions in these inputs affected resource use and emergy-based sustainability indicators. Under the enhanced cooking efficiency (ECE) scenario, three categories of measures—integrated cooking practices, intelligent power regulation, and waste-heat recovery—were implemented during cooking. Specifically, stand-alone high-temperature frying was replaced with combined cooking techniques such as batch steaming; power was modulated during stewing and braising, while idle heating was minimized; pre-soaking and cascaded waste-heat utilization practices, including water preheating and heat retention, were adopted; and prolonged heating was replaced with pressure cooking. These interventions were represented in the model by a 28% reduction in cooking-energy consumption. Under the combined optimization scenario (COS), all five interventions specified under ICLR, EMEE, TAAS, MOSR, and ECE were implemented concurrently.

### 2.5. Data Sources

Dish-level data, cooking durations, and the recommended number of consumers per dish were obtained from the Chinese cookbook compiled by Chen and Fang [20] and an online recipe platform [21]. The rates of natural gas and electricity consumption at different heat settings (high, medium, and low) were derived from Liu et al. [6]. Agricultural production input data for all ingredients were obtained from the *National Compilation of Cost-Benefit Data for Agricultural Products* (2025) [51]. Energy price data were obtained from the *China Electric Power Statistical Yearbook* (2025) [52] and the *China Price Statistical Yearbook* (2025) [53]. Net topsoil loss rates were adopted from Wu et al. [54]. Solar radiation and wind speed data were obtained from the *China Wind and Solar Energy Resources Bulletin* (2025) [55], while precipitation data were obtained from the *China Water Statistical Yearbook* (2025) [56]. Terrestrial heat-flow data were obtained from the compilation of heat flow data in the continental area of China [57,58]. The energy conversion coefficients used to express selected material inputs in joules (J) were obtained from the second edition of *Agroecology* [59].

## 3. Results

### 3.1. Emergy Input Structure

**Figure 2:**
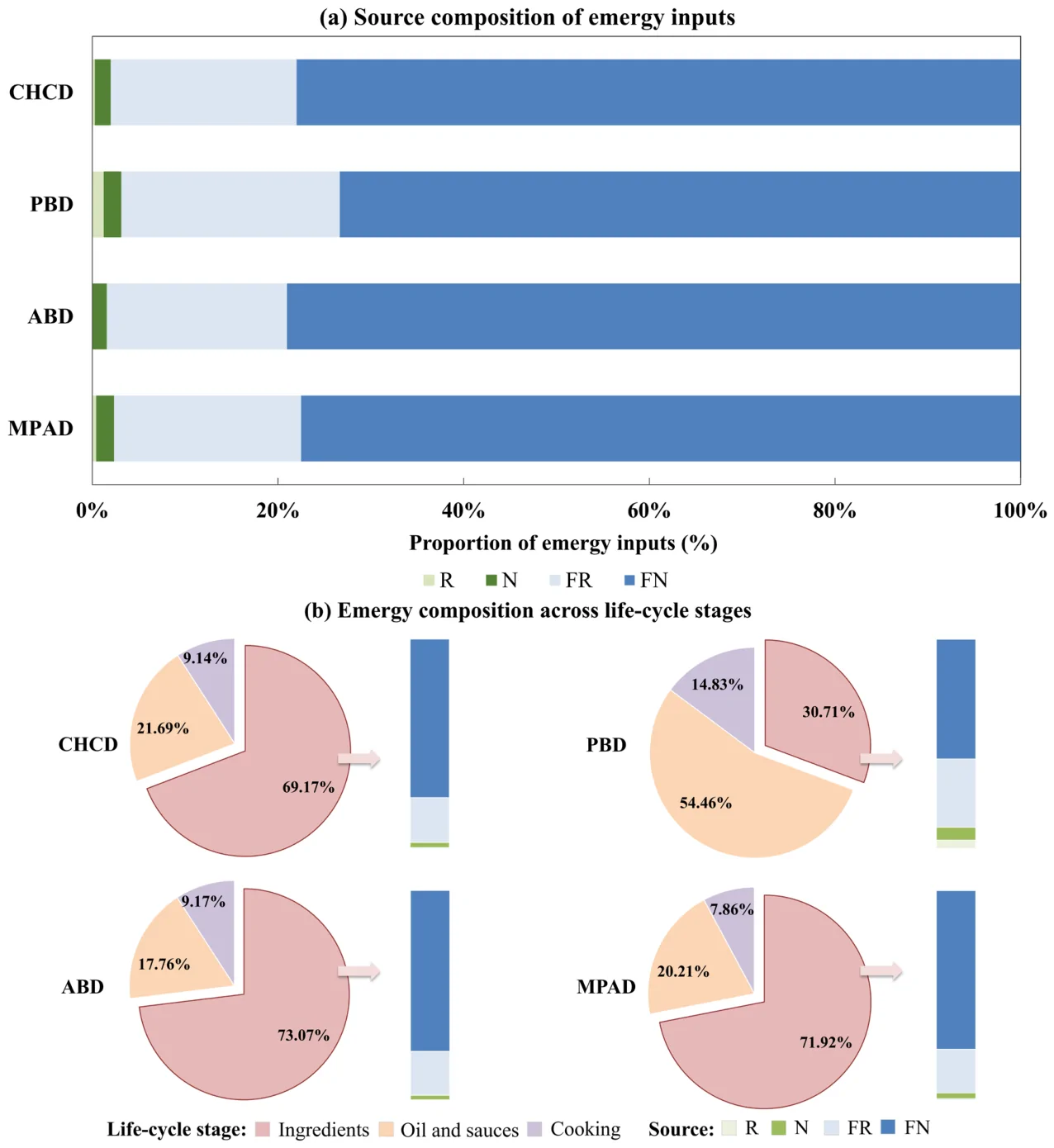
Emergy input structures of the full Chinese home-cooked dish (CHCD) sample and the three dish categories: (**a**) source composition of total emergy inputs; (**b**) emergy composition across life-cycle stages and source categories. PBD, plant-based dishes; ABD, animal-based dishes; MPAD, mixed plant–animal dishes; R, renewable natural emergy; N, nonrenewable natural emergy; FR, renewable purchased emergy; FN, nonrenewable purchased emergy.

The total emergy input to the CHCD system was 5.93 × 10^12^ sej·dish^−1^, and its input structure was characterized by a high dependence on the socioeconomic system. Renewable natural emergy (R), nonrenewable natural emergy (N), renewable purchased emergy (FR), and nonrenewable purchased emergy (FN) accounted for 0.27%, 1.71%, 20.04%, and 77.99% of the total input, respectively. Purchased emergy inputs (5.82 × 10^12^ sej·dish^−1^) were approximately 50 times the natural emergy inputs (1.17 × 10^11^ sej·dish^−1^), indicating that the emergy demand of the CHCD system was met almost entirely by the economic system, with only limited direct use of local environmental resources. In terms of renewability, the nonrenewable emergy input (4.73 × 10^12^ sej·dish^−1^) was 3.93 times the renewable emergy input (1.20 × 10^12^ sej·dish^−1^), reflecting a high dependence on nonrenewable resources. As shown in Figure 2a, FN dominated the input structure of all three dish categories, although their total emergy inputs differed substantially. ABD had the highest total emergy input (9.87 × 10^12^ sej·dish^−1^), approximately six times that of PBD (1.64 × 10^12^ sej·dish^−1^), whereas MPAD had an intermediate value (5.94 × 10^12^ sej·dish^−1^) that was nearly identical to the overall CHCD mean. ABD had not only the highest total emergy input but also the highest nonrenewable emergy input, followed by MPAD and PBD.

Across the life-cycle stages (Figure 2b), ingredients accounted for the largest share of the CHCD emergy input, at 69.17% (4.11 × 10^12^ sej·dish^−1^), followed by oil and sauces at 21.69% (1.29 × 10^12^ sej·dish^−1^) and cooking at 9.14% (5.42 × 10^11^ sej·dish^−1^). Within the ingredient stage, R, N, FR, and FN accounted for 0.38%, 2.47%, 21.40%, and 75.75% of the stage total, respectively. Notably, the natural emergy embodied in ingredients (1.17 × 10^11^ sej·dish^−1^) was equivalent to the total natural emergy input to the CHCD system, indicating that the emergy inputs associated with oil, sauces and cooking were supplied almost entirely through economic feedback. The composition of emergy inputs differed markedly among dish categories. PBD exhibited the most distinctive profile, with the lowest ingredient emergy input (5.02 × 10^11^ sej·dish^−1^). Consequently, emergy inputs from oil and sauces accounted for the dominant share (54.46%), surpassing the contribution from ingredients, in sharp contrast to the ingredient-dominated pattern observed in the other categories. The ingredient stage of PBD also had the highest proportions of natural and renewable emergy among all dish categories. ABD had the highest emergy inputs from ingredients (7.22 × 10^12^ sej·dish^−1^), oil and sauces (1.75 × 10^12^ sej·dish^−1^), and cooking (9.06 × 10^11^ sej·dish^−1^), as well as the highest FN input in the ingredient stage (5.55 × 10^12^ sej·dish^−1^), and was characterized by both high emergy consumption and a high proportion of non-renewable emergy. MPAD most closely resembled the overall CHCD input structure: its ingredient input (4.27 × 10^12^ sej·dish^−1^) was slightly above the mean, whereas its inputs from oil and sauces (1.20 × 10^12^ sej·dish^−1^) and cooking (4.67 × 10^11^ sej·dish^−1^) were slightly below the corresponding means.

### 3.2. Agricultural Inputs and Environmental Loading Among Ingredients

**Figure 3:**
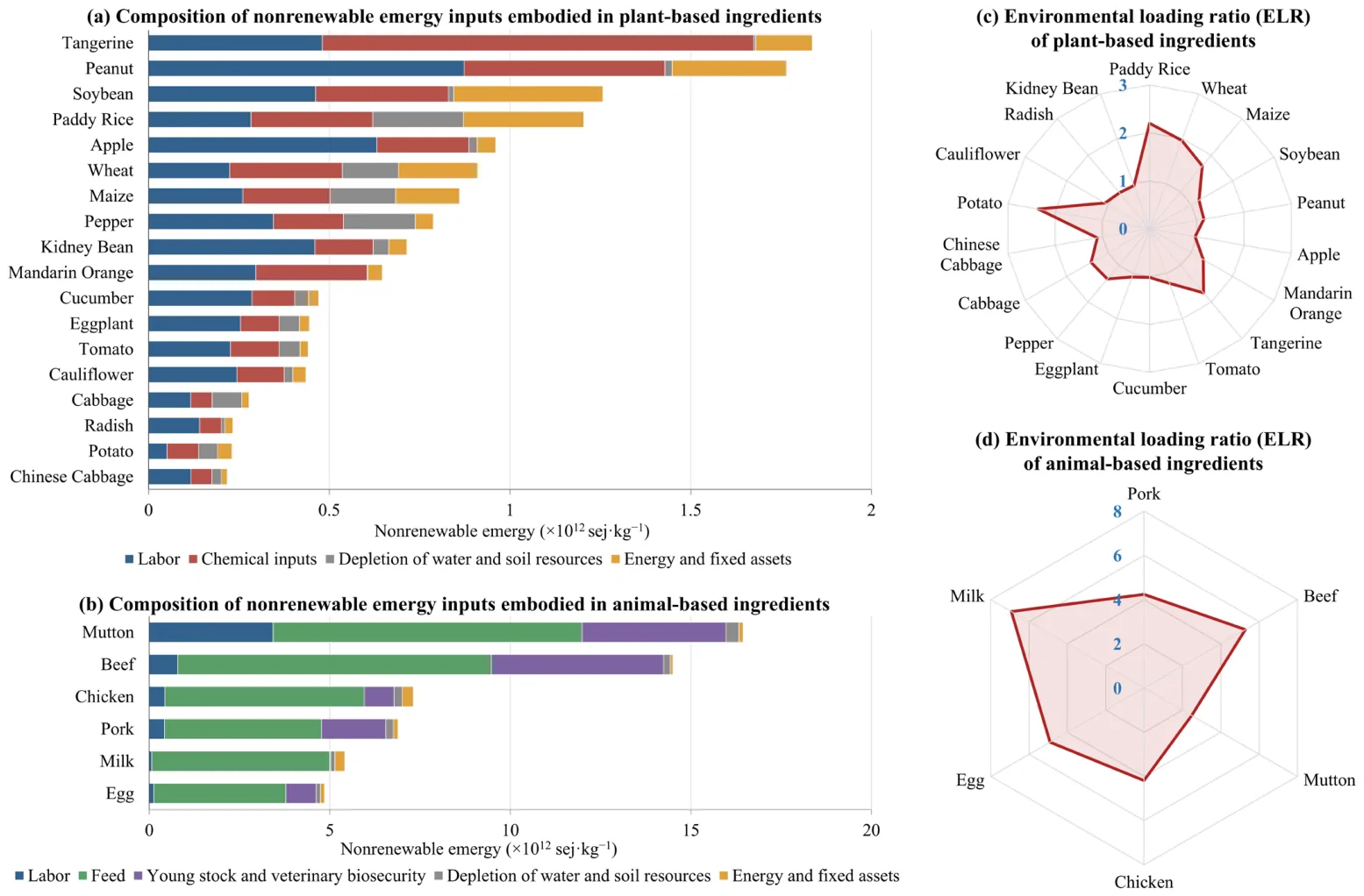
Nonrenewable agricultural emergy inputs and environmental loading of ingredients: (**a**) composition of nonrenewable emergy inputs embodied in plant-based ingredients; (**b**) composition of nonrenewable emergy inputs embodied in animal-based ingredients; (**c**) environmental loading ratio of plant-based ingredients; (**d**) environmental loading ratio of animal-based ingredients.

During the agricultural production stage, the mass-specific nonrenewable emergy embodied in animal-based ingredients was substantially higher than that embodied in plant-based ingredients. As shown in Figure 3a,b, mass-specific nonrenewable emergy inputs were generally several-fold higher for animal-based ingredients than for plant-based ingredients. Among animal-based ingredients, mutton had the highest value (16.45 × 10^12^ sej·kg^−1^), whereas Chinese cabbage had the lowest value among plant-based ingredients (0.22 × 10^12^ sej·kg^−1^), representing an approximately 75-fold difference. Even eggs, which had the lowest value among animal-based ingredients (4.86 × 10^12^ sej·kg^−1^), exceeded tangerine, which had the highest value among plant-based ingredients (1.84 × 10^12^ sej·kg^−1^). For plant-based ingredients, nonrenewable agricultural inputs were dominated by labor and chemical inputs, reflecting low levels of mechanization and a high reliance on fertilizers and pesticides. Different ingredient categories exhibited distinct emergy input profiles. Chemical inputs dominated fruit production, including tangerines and mandarin oranges, accounting for up to 65% in tangerine production. Cereals and oil crops, including paddy rice, wheat, and soybeans, showed higher contributions from energy and fixed assets, as well as from water and soil depletion, indicating greater irrigation and energy demands. Most vegetables, by contrast, were dominated by labor inputs and generally had lower total nonrenewable emergy inputs. For animal-based ingredients, feed was the single largest contributor to nonrenewable agricultural inputs across all animal products, accounting for 52.03–91.18% of their total inputs. In terms of total nonrenewable emergy inputs, ruminant meats, including mutton and beef, had higher values, whereas eggs had the lowest.

The environmental loading ratio (ELR) further quantified the relative pressure exerted on the local ecosystem by the production of different ingredients. As shown in Figure 3c,d, the ELR values of animal-based ingredients (2.49–6.92) were generally higher than those of plant-based ingredients (0.96–2.36). Among animal-based ingredients, milk had the highest ELR (6.92), followed by beef (5.29) and eggs (4.90), whereas mutton had the lowest ELR (2.49). Among plant-based ingredients, potatoes and paddy rice had relatively high ELR values of 2.36 and 2.20, respectively. Most vegetables, including radishes, cauliflowers, and cucumbers, as well as legumes, generally had lower values. Kidney beans had the lowest ELR (0.96). Notably, the ELR was not directly proportional to the absolute magnitude of nonrenewable emergy input. Potatoes provided a representative example: despite having one of the lowest total nonrenewable emergy input values (0.23 × 10^12^ sej·kg^−1^), they had the highest ELR because their renewable emergy base (R + FR) was only 0.10 × 10^12^ sej·kg^−1^. Mutton exhibited the opposite pattern: it had the largest renewable emergy base among animal-based ingredients (R + FR, 6.61 × 10^12^ sej·kg^−1^) and the lowest ELR.

### 3.3. Emergy-Based Sustainability Evaluation and Spatial Heterogeneity

**Figure 4:**
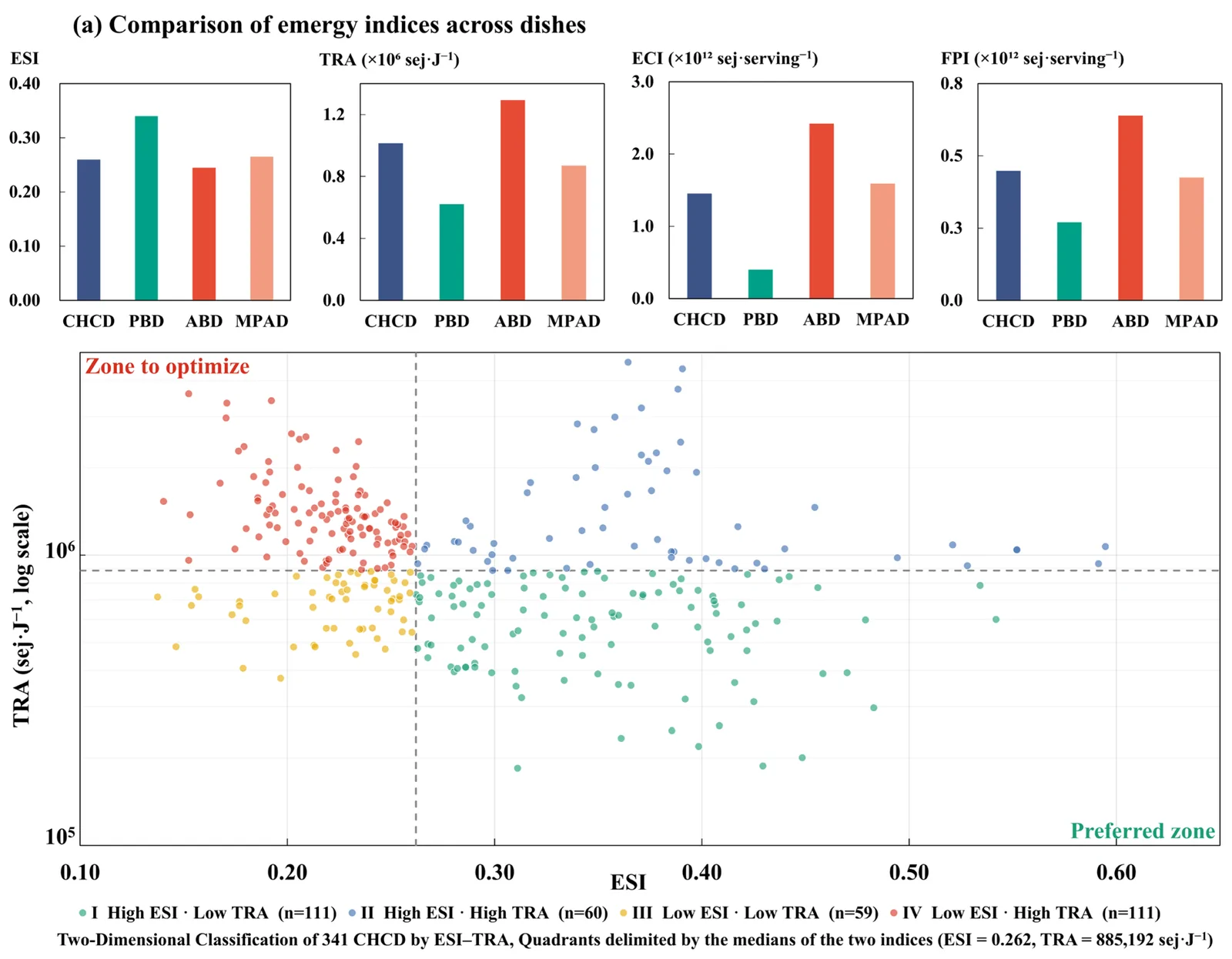

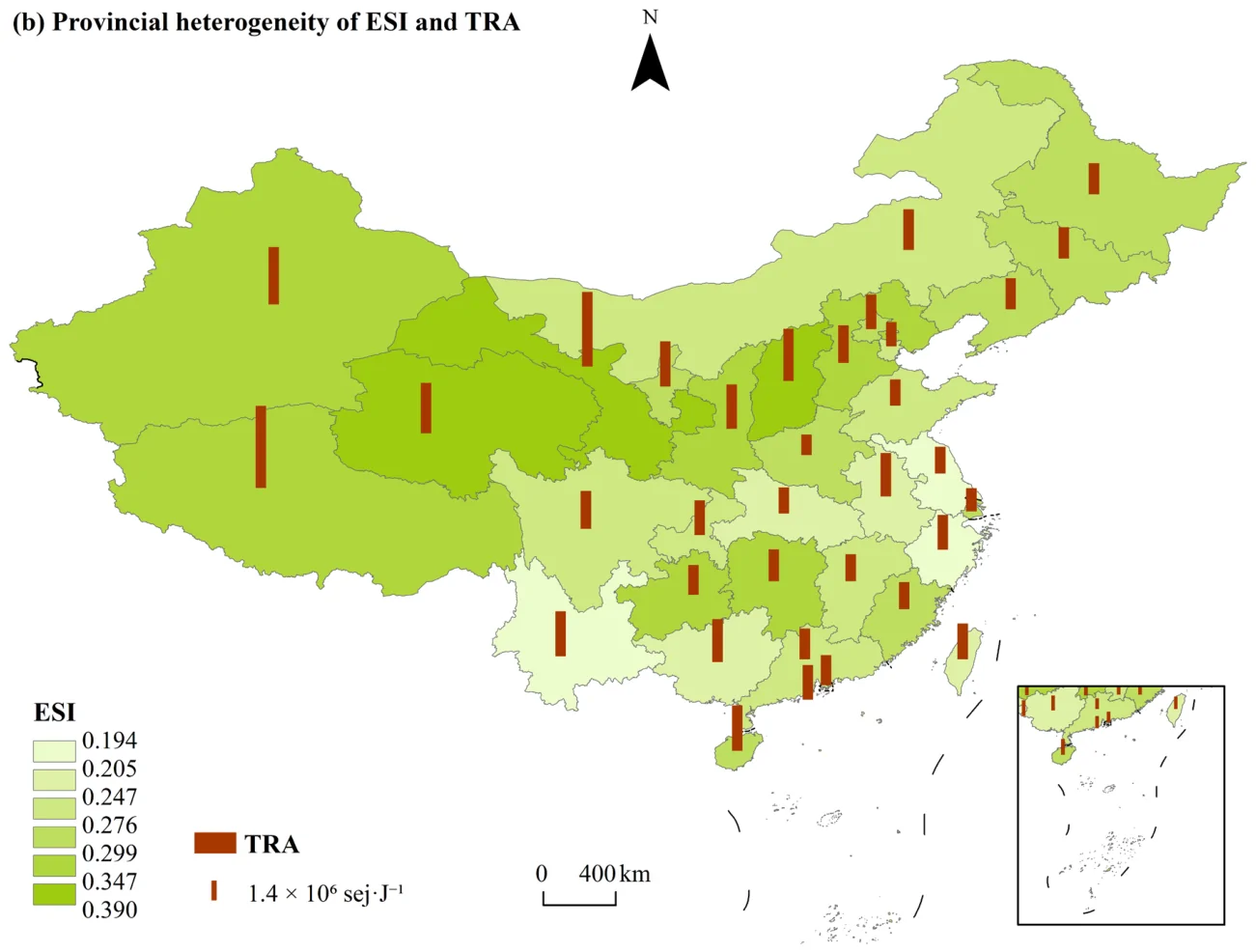
Emergy-based sustainability of Chinese home-cooked dishes (CHCDs) and its spatial heterogeneity: (**a**) comparison of emergy sustainability index (ESI), transformity (TRA), emergy consumption intensity (ECI), and food processing intensity (FPI) for the full CHCD sample and the three dish categories, together with the two-dimensional classification of 341 CHCDs based on ESI and TRA; (**b**) provincial heterogeneity in ESI and TRA. PBD, plant-based dishes; ABD, animal-based dishes; MPAD, mixed plant–animal dishes.

The emergy-based indicators of CHCDs varied with ingredient composition and cooking method. Across the 341 CHCDs, the mean emergy sustainability index (ESI) was 0.26, while the mean transformity (TRA), emergy consumption intensity (ECI), and food processing intensity (FPI) values were 1.02 × 10^6^ sej·J^−1^, 1.45 × 10^12^ sej·serving^−1^, and 4.64 × 10^11^ sej·serving^−1^, respectively. As shown in Figure 4a, PBD had the highest ESI (0.34) and the lowest TRA (6.22 × 10^5^ sej·J^−1^), ECI (4.00 × 10^11^ sej·serving^−1^), and FPI (2.82 × 10^11^ sej·serving^−1^). ABD exhibited the opposite pattern, with the lowest ESI (0.24) and the highest TRA (1.29 × 10^6^ sej·J^−1^), ECI (2.42 × 10^12^ sej·serving^−1^), and FPI (6.53 × 10^11^ sej·serving^−1^). Compared with ABD, MPAD had a higher ESI (0.27) and markedly lower TRA (8.70 × 10^5^ sej·J^−1^) and ECI (1.59 × 10^12^ sej·serving^−1^), while its FPI (4.58 × 10^11^ sej·serving^−1^) was close to the overall sample mean. The two-dimensional classification based on ESI and TRA further revealed pronounced polarization in environmental sustainability and emergy efficiency among the dishes. The preferred zone, characterized by high ESI and low TRA, and the optimization-priority zone, characterized by low ESI and high TRA, each contained 111 dishes. However, their category compositions differed substantially. The optimization-priority zone was dominated by ABD (63%; 70 of 111 dishes), whereas PBD and MPAD together accounted for 97% of the preferred zone. Furthermore, dishes in the optimization-priority zone generally exhibited elevated ECI and FPI values, reflecting greater resource costs associated with satisfying consumer demand and a higher degree of food processing.

In terms of geographic origin and cuisine type, both ESI and TRA exhibited a distinct declining spatial gradient from the northwestern inland regions toward the southeastern coast at the provincial level. As shown in Figure 4b, regions south of the Yangtze River were generally characterized by low ESI and TRA values, whereas northwestern regions had high values for both indicators. Among provincial-level administrative regions with sufficient sample sizes (*n* ≥ 7), Shaanxi, Hunan, Beijing, Fujian, Shandong and Shanghai exhibited relatively favorable ESI–TRA profiles. Shanghai, Fujian, and Shandong combined higher ESI with lower TRA and were therefore positioned closest to the preferred zone. By contrast, Zhejiang, Anhui, and Taiwan simultaneously had low ESI and high TRA and were positioned closer to the optimization-priority zone.

The eight major traditional Chinese cuisines (*n* = 146) represented the largest share of the CHCD sample and differed substantially in environmental sustainability. In terms of ESI, Hunan cuisine (0.32) and Fujian cuisine (0.30) had relatively high values, followed by Sichuan cuisine (0.28) and Shandong cuisine (0.27); all four values exceeded the median of the overall CHCD sample. By contrast, Zhejiang cuisine (0.19) and Jiangsu cuisine (0.21) had lower ESI values. In terms of TRA, Shandong cuisine (8.60 × 10^5^ sej·J^−1^), Jiangsu cuisine (8.80 × 10^5^ sej·J^−1^), and Fujian cuisine (9.00 × 10^5^ sej·J^−1^) had the lowest values, indicating higher emergy efficiency. Anhui cuisine (1.43 × 10^6^ sej·J^−1^), Sichuan cuisine (1.25 × 10^6^ sej·J^−1^), and Zhejiang cuisine (1.15 × 10^6^ sej·J^−1^) had higher TRA values, indicating lower emergy efficiency. Among the eight major cuisines, only Shandong and Jiangsu cuisines had TRA values below the median of the overall CHCD sample. Considering both indicators, Shandong cuisine combined high ESI with low TRA and aligned most closely with the profile of the preferred zone. By contrast, Zhejiang and Anhui cuisines had the highest proportions of dishes classified in the optimization-priority zone.

### 3.4. Comparative Scenario Assessment of CHCD Optimization

To identify effective pathways and underlying mechanisms for the sustainable transformation of the CHCD system, this study quantified the responses of emergy-based indicators to five standalone optimization measures and a combined optimization scenario relative to the business-as-usual (BAU) scenario. The effects of the standalone measures varied substantially in direction and magnitude across indicators. Each measure targeted a distinct aspect of system optimization. Integrated crop–livestock recycling (ICLR) predominantly enhanced system sustainability; technological advancement and appropriate scaling-up (TAAS) moderately increased resource-use efficiency and reduced environmental loading; moderate oil and sauce reduction (MOSR) and enhanced cooking efficiency (ECE) more directly reduced inputs associated with food processing and consumption; and enhanced mechanization and energy efficiency (EMEE) had a relatively marginal overall effect. By contrast, the combined optimization scenario (COS) produced the most comprehensive improvements across all four indicators.

**Figure 5:**
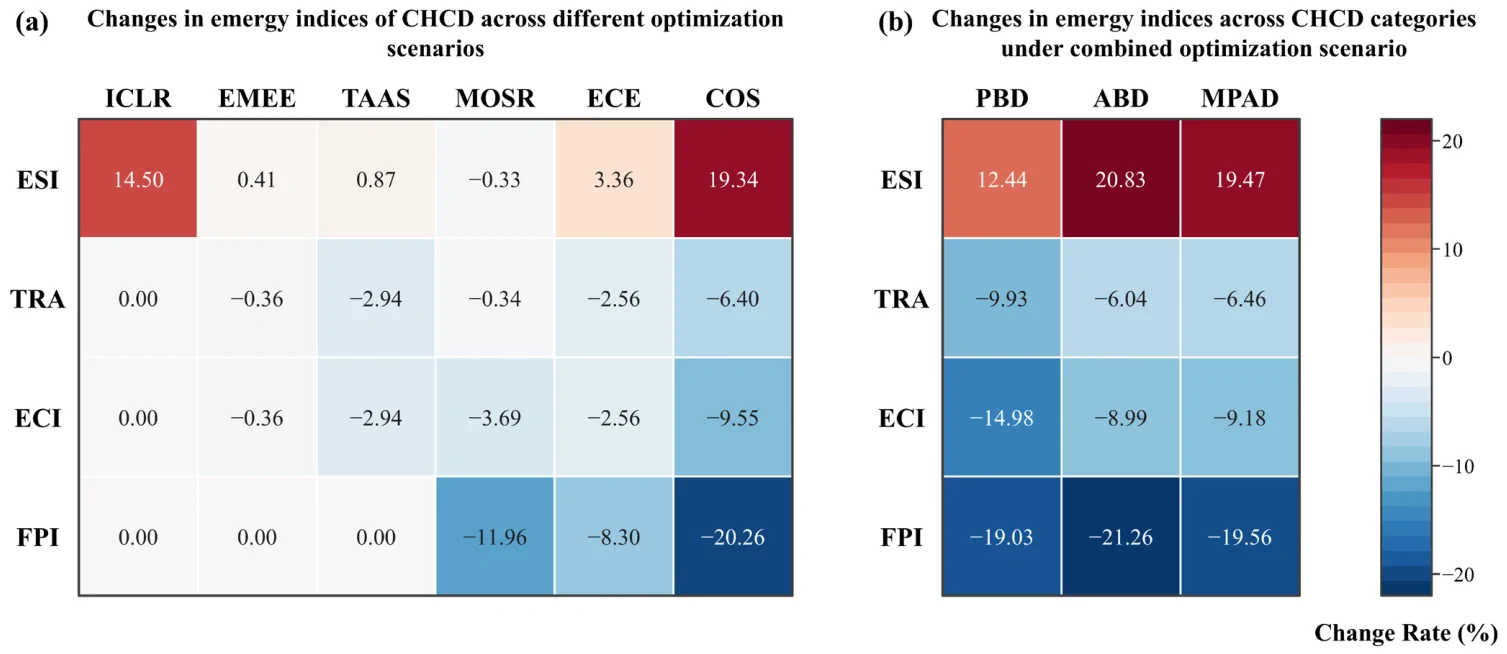
Percentage changes in emergy-based indices relative to the business-as-usual scenario: (**a**) system-level changes under five standalone scenarios and COS; (**b**) dish-category-level changes under COS. CHCD, Chinese home-cooked dishes; PBD, plant-based dishes; ABD, animal-based dishes; MPAD, mixed plant–animal dishes; ESI, emergy sustainability index; TRA, transformity; ECI, emergy consumption intensity; FPI, food processing intensity; ICLR, integrated crop–livestock recycling; EMEE, enhanced mechanization and energy efficiency; TAAS, technological advancement and appropriate scaling-up; MOSR, moderate oil and sauce reduction; ECE, enhanced cooking efficiency; COS, combined optimization scenario.

As shown in Figure 5a, among the standalone measures, ICLR produced the largest increase in the ESI of the CHCD system, raising it by 14.50%. ECE increased ESI by 3.36%, whereas EMEE and TAAS produced smaller increases of 0.41% and 0.87%, respectively. Under COS, ESI increased by 19.34%. TRA and ECI decreased under several optimization pathways. TAAS reduced both indicators by 2.94%, ECE reduced both by 2.56%, and MOSR reduced TRA and ECI by 0.34% and 3.69%, respectively. Under COS, TRA and ECI reached their lowest values of 9.50 × 10^5^ sej·J^−1^ and 1.32 × 10^12^ sej·serving^−1^, respectively. For FPI, MOSR and ECE produced reductions of 11.96% and 8.30%, respectively, whereas COS produced the largest reduction of 20.26%.

The effects of the optimization measures varied markedly among dish categories, with Figure 5b further illustrating the category-specific responses of PBD, ABD, and MPAD to COS. Under the full implementation of all measures in COS, ABD exhibited the largest relative increase in ESI (20.83%), followed by MPAD (19.47%) and PBD (12.44%). TRA and ECI improved consistently across all three dish categories. PBD showed the greatest potential to enhance production efficiency and reduce consumption intensity, with reductions of 9.93% in TRA and 14.98% in ECI, respectively. In absolute terms, however, ABD had the largest reduction in TRA, whereas MPAD had the largest reduction in ECI. ABD also had the largest percentage reduction in FPI (21.26%), followed by MPAD (19.56%) and PBD (19.03%).

## 4. Discussion

### 4.1. Emergy-Based Sustainability Analysis of the CHCD System

Emergy analysis provides a comprehensive and systematic framework for evaluating the ecological sustainability of the CHCD system [13]. Compared with assessment approaches that focus primarily on carbon footprints or direct energy use, emergy accounting standardizes heterogeneous inputs—including those associated with primary agricultural production, industrial food processing, culinary condiments, and household cooking energy—on a common solar emergy basis [60]. This approach enables the integrated quantification of both the natural environmental support embodied in these inputs and socioeconomic feedbacks throughout the supply chain [61]. Our findings indicate that each dish should not be regarded solely as a passive consumption unit defined by ingredient quality or caloric density; instead, it represents a complex socioecological subsystem embedded within broader industrial networks.

The dominance of FN (77.99%), together with a purchased-to-natural emergy input ratio of approximately 50:1, indicates that household food consumption depends strongly on socioeconomic inputs and makes only limited direct use of local environmental resources. This pattern is consistent with the broader evolution of modern agri-food systems [9], in which progressive commodification and energy intensification shift dependence from local ecosystem-service flows toward market-supplied inputs, including fertilizers, fossil fuels, labor, and infrastructure. Although such systems may increase food-provisioning capacity at the consumption stage, they may also reduce resilience to market volatility, including fuel-price shocks and supply-chain disruptions [62,63]. Accordingly, the low emergy sustainability observed in the CHCD system did not arise from any single stage but reflected the cumulative dominance of nonrenewable inputs across the production–processing–cooking continuum without a commensurate renewable emergy base.

Furthermore, the FPI results provide a complementary perspective on the contribution of culinary preparation to the resource intensity of the CHCD system. Higher FPI values reflected greater per-serving emergy inputs associated with culinary oils, sauces, and energy-intensive cooking methods, such as stewing and red braising, which are used to enhance palatability and texture [64,65]. This pattern suggests that environmental burdens are not only inherited from upstream supply chains but are also compounded during downstream culinary preparation. Overall, resource intensification across the supply chain suggests that dietary analyses focused on raw agricultural commodities may underestimate the full environmental and socioeconomic resource requirements of actual household consumption, thereby demonstrating the methodological value of using the dish as the fundamental accounting unit.

Ingredient composition was a major source of variation in emergy inputs among CHCDs. The approximately sixfold difference between ABD and PBD was consistent with cumulative amplification along the emergy transformation hierarchy. Animal production involves multistage energy conversions, extending from fossil-energy and fertilizer inputs through feed cultivation to livestock production. At each successive transformation, available energy is dissipated while emergy becomes increasingly concentrated, resulting in higher solar transformity at higher trophic levels. Consequently, even when providing comparable or greater dietary energy, ABD required substantially greater upstream emergy support. This pattern was most pronounced for ruminant and other feed-intensive livestock products, whose emergy profiles reflected the combined contributions of feed cultivation, veterinary management, and farm operations and were associated with higher TRA and ECI values in the corresponding dishes.

Nevertheless, the relative advantage of plant-based ingredients did not make PBD inherently low-burden. Within PBD, the emergy contribution of edible oils and compound sauces exceeded that of the primary plant ingredients and became the dominant component. This finding indicates that seasoning practices can substantially alter the overall emergy input structure and sustainability performance of a dish because edible oils and sauces are industrially refined commodities that embody emergy inputs from both primary agricultural production and downstream processing.

The contrasting ELR patterns of potatoes and mutton (Figure 3c,d) further show that total nonrenewable emergy input and relative environmental loading are not directly proportional. Because ELR depends on both nonrenewable inputs and the renewable emergy base, these two measures capture complementary dimensions of ingredient-related environmental performance and should be considered together when evaluating ingredient choices. Furthermore, although some sustainable-diet assessments classify vegetables and cereals together as low-impact plant foods [66], our findings showed that cereal-based dishes had higher ELR values than vegetable-based dishes. In addition to the lower nonrenewable emergy inputs associated with fresh vegetable production, one contributing mechanism was the difference in culinary conversion pathways. Vegetables were predominantly used as minimally processed primary ingredients, whereas cereals in CHCDs were frequently processed into secondary ingredients, such as noodles, vermicelli, and dumpling wrappers. Across the 188 secondary ingredients identified in this study, mechanical processing, thermal inputs, and processing-related energy losses added further resource requirements, and each additional transformation could increase unit emergy intensity. This finding highlights a limitation of sustainable-diet assessments based solely on an animal-based versus plant-based distinction: the environmental performance of a dish depends not only on its principal raw ingredients but also on culinary processing intensity and culturally specific dietary practices.

### 4.2. Spatial Heterogeneity in Emergy-Based Indicators of the CHCD System

Regional variation in the sustainability of CHCDs may reflect differences in local dietary culture, ingredient preferences, and cooking methods. At the provincial scale, both ESI and TRA showed a declining gradient from inland northwestern China toward the southeastern coast. Because lower ESI indicates poorer sustainability whereas lower TRA indicates higher emergy efficiency, this common numerical gradient represents a spatial trade-off rather than a uniform decline in system performance. The spatial pattern was consistent with regional differences in dish composition. Northwestern dishes made greater use of ingredients with high emergy inputs, such as mutton. This northwestern pattern may also reflect the substantial renewable resource base associated with grassland-based livestock systems. Southern dishes, by contrast, frequently involved refined ingredients and complex cooking procedures and showed greater dependence on nonrenewable emergy inputs, including chemical inputs associated with ingredient production. By comparison, northern and northeastern China exhibited more balanced ESI–TRA profiles. MPAD predominated in both regions, while poultry and pork were the principal animal-based ingredients. Both regions also had relatively low overall FPI values despite having higher proportions of fried dishes than other regions.

Beyond dishes consumed nationwide (e.g., stir-fried tomato with egg), the eight major traditional Chinese cuisines (*n* = 146) capture substantial geographic and culinary diversity in Chinese household diets. ESI values decreased in the following order: Hunan, Fujian, Sichuan, Shandong, Cantonese, Anhui, Jiangsu, and Zhejiang. This pattern was broadly consistent with previous footprint-based research [9], which classified Sichuan, Hunan, and Shandong cuisines as low-burden; Cantonese and Jiangsu cuisines as moderate-burden; and Zhejiang and Anhui cuisines as high-burden. The present analysis extends this comparison by incorporating labor, energy, and fixed-asset inputs.

Based on TRA, emergy efficiency ranked from highest to lowest as follows: Shandong, Jiangsu, Fujian, Cantonese, Hunan, Zhejiang, Sichuan, and Anhui. Shandong was the only cuisine with both an ESI above the overall sample mean and a TRA below the overall sample mean, indicating a favorable balance between sustainability and emergy efficiency. This pattern may reflect the relatively balanced proportions of cereals, vegetables, and meat in Shandong cuisine, together with a savory–salty seasoning profile associated with lower use of edible oils, refined sugar, and compound sauces. This interpretation is consistent with recent carbon-accounting research reporting the lowest production- and cooking-related carbon emissions per unit mass for Shandong cuisine [65].

Two features may help explain the lower estimated emergy efficiency of some regional cuisines. The first is limited diversity within the sampled dishes combined with a greater prevalence of secondary processing, as illustrated by the fermentation of tofu into hairy tofu in Anhui cuisine and the curing of pork into preserved meat and sausages in Sichuan cuisine. The second is the extensive use of red meat and condiments, including broad bean paste. Despite their lower emergy efficiency, Hunan and Sichuan cuisines retained relatively high overall sustainability. This contrast may be associated with their reliance on lower-burden vegetable inputs, such as peppers, and the prevalence of short-duration thermal processing. Rapid stir-frying, a major cooking technique in Hunan and Sichuan cuisines, can reduce fuel consumption relative to the prolonged stewing and braising common in Zhejiang and Cantonese cuisines [67]. This finding is consistent with the reported ranking of cooking-method energy efficiency: rapid stir-frying, steaming, deep-frying, and stewing [6]. Across the eight cuisines, four cross-cuisine intervention points emerged: using locally advantageous crops, adopting short-duration heating, maintaining ingredient diversity, and minimizing secondary-ingredient processing. These principles provide a basis for developing context-specific and ecologically aligned agri-food sustainability strategies in China.

### 4.3. Pathways Toward a Sustainable CHCD System

Our scenario simulations revealed that, although each optimization pathway moved the CHCD system toward greater sustainability, changes in the key emergy indicators indicated that standalone interventions had limited system-wide effects. Rather, the sustainable transition of the CHCD system requires coordinated structural adjustments that combine improvements in upstream production efficiency with downstream consumption-side measures across the value chain. This finding highlights the need to examine supply- and demand-side intervention mechanisms from a systems perspective and provides a basis for identifying pathways toward CHCD sustainability.

The optimization measures exhibited pronounced indicator-specific effects. Integrated crop–livestock recycling (ICLR) primarily enhanced system sustainability by altering the system’s renewability profile and increasing the renewable shares of concentrate feed and electricity inputs, thereby substantially increasing ESI. Because ESI is calculated as the ratio of EYR to ELR, this increase reflected a more favorable balance between emergy yield and environmental loading rather than a reduction in the total magnitude of external inputs. This mechanism also accounts for the negligible changes in TRA, ECI, and FPI under ICLR. By contrast, technological advancement and appropriate scaling-up (TAAS) produced concurrent and balanced reductions in TRA and ECI. These reductions resulted from greater efficiency in the use of fertilizers and pesticides and greater feed-use efficiency, consistent with the measure’s objective of reducing material inputs. Enhanced mechanization and energy efficiency (EMEE) produced only marginal improvements (<0.42%) across all indicators, indicating that EMEE alone has limited potential to improve the overall performance of the CHCD system under its current emergy input structure. This result is consistent with a common pattern identified in agri-food emergy assessments: improvements in on-farm energy efficiency may have limited system-level effects when emergy-intensive upstream inputs, particularly agrochemicals and commercial feed, dominate total emergy use [68]. This interpretation is consistent with previous emergy assessments of Chinese crop production [69] and indicates that localized technical upgrades will remain limited in their system-wide effectiveness unless structural dependence on emergy-intensive upstream inputs is also reduced.

Consumption-side interventions were particularly effective in reducing FPI. Both moderate oil and sauce reduction (MOSR) and enhanced cooking efficiency (ECE) reduced food processing intensity by lowering the material and energy requirements of dish preparation: MOSR reduced oil and sauce inputs, whereas ECE reduced cooking-energy demand. MOSR may therefore help address the high oil and sauce inputs associated with some dishes, while the two measures together identify dietary adjustment and cooking-efficiency improvement as complementary consumption-side interventions. However, MOSR involved an indicator-level trade-off: although it reduced ECI and FPI, it also caused a slight decline in ESI (0.33%). This result indicates that consumption-side reductions in material use alone may be insufficient to improve overall ecological sustainability and may need to be complemented by measures that increase the use of renewable resources. By contrast, ECE simultaneously reduced FPI, TRA, and ECI while increasing ESI. ECE therefore represents a potentially effective modeled intervention capable of delivering concurrent improvements in resource-use efficiency and environmental performance.

Integrating interventions throughout the value chain produced more comprehensive improvements than implementing individual measures in isolation, making the combined optimization scenario (COS) the best-performing scenario among those evaluated for the transformation of the CHCD system. COS achieved the most favorable performance across all indicators. In particular, the increase in ESI under COS (19.34%) slightly exceeded the summed increases achieved by the standalone interventions (18.81%), representing a 0.53-percentage-point superadditive effect in the scenario results. This pattern may reflect interactions between upstream improvements in resource circularity and input-use efficiency and downstream reductions in material and cooking-energy use. Specifically, supply-side measures increased the renewable share of external emergy inputs, whereas demand-side measures reduced the requirements for cooking energy and other material inputs. Their joint implementation amplified the overall improvement in sustainability, supporting the prioritization of coordinated interventions across the production–consumption continuum.

Implementing sustainability pathways for the CHCD system requires differentiated strategies tailored not only to the resource-use profiles of dish categories but also to their culinary contexts. ABD showed the largest increase in ESI and the greatest reduction in FPI, which may reflect its initially low renewability and greater potential to benefit from ICLR and reductions in cooking-related inputs. MOSR was modeled as a moderate adjustment aimed at reducing the emergy inputs associated with edible oil and sauces. Nevertheless, these modeled effects should be interpreted as context-dependent options rather than uniform prescriptions, because regional dietary characteristics and behavioral heterogeneity can substantially influence both optimization outcomes and the practical applicability of dietary interventions [70]. This consideration is particularly relevant to Chinese home cooking, as standardized dietary frameworks may not fully capture variations in food culture and public acceptability [46]. Chinese home-cooked dishes are embedded in diverse regional culinary traditions, in which ingredient combinations, seasoning intensity, and cooking techniques help define characteristic flavors, textures, and culinary identity [8]. Consequently, the practical acceptability of MOSR, ECE, or a potential shift from ABD toward MPAD identified from an emergy perspective may vary across regional cuisines and dish types. Uniform reductions in oil and sauces or direct ingredient substitution could alter the sensory profiles and perceived authenticity of some dishes. Culturally compatible implementation should therefore prioritize incremental and cuisine-specific adjustments, such as reducing condiments where they are not central to dish identity, improving cooking-energy efficiency while retaining established techniques, and favoring MPAD combinations already present in local culinary repertoires. These pathways represent potential resource-efficiency implications of the model rather than dietary recommendations. Consumer preferences, willingness to change eating habits, economic considerations, and actual household behavior were not assessed; therefore, their practical applicability requires further validation in specific regional and household contexts.

### 4.4. Limitations and Future Research

Several limitations should be considered when interpreting the results of this study. First, the dish-level data were derived primarily from standardized recipes rather than direct observations of household cooking. Actual households may differ in ingredient selection and quantities, portion sizes, and cooking practices. Although the 341 dishes covered 34 provincial-level administrative regions, the dataset may not directly reflect the popularity and consumption frequency of the dishes. Moreover, the reported regional origin of a dish represents its culinary origin, which shapes its ingredient combinations and cooking techniques, but does not necessarily correspond to the actual agricultural production locations of its ingredients.

Second, serving sizes were estimated from the recommended number of consumers specified in the recipe sources. Actual portion sizes may vary according to meal structure, individual appetite, and dining practices, thereby affecting the per-serving results. Cooking-energy consumption was also estimated using recipe-based cooking durations and published energy-consumption rates rather than direct household measurements. These standardized assumptions improve comparability among dishes but may not fully represent the variability of real-world cooking. Third, although literature-based UEVs were harmonized to a common emergy baseline, they remain subject to uncertainty associated with differences in production technologies, regional conditions, and data years. The UEVs estimated for cooking oil and sauces also depend on assumptions about raw-material composition, processing yields, and processing energy requirements. These uncertainties, together with those associated with assumptions about ingredient mass and cooking-energy consumption, represent a methodological limitation of this study. Future research should address this important limitation through more comprehensive data collection and refined estimation of emergy parameters for food systems, particularly the UEVs of cooking oil and sauces.

Finally, the optimization scenarios represent model-based interventions rather than measures validated in actual households. Their practical effectiveness may depend on cooking skills, economic costs, household preferences, and the cultural and sensory acceptability of changes to ingredients and cooking practices. The projected improvements should therefore be interpreted as potential system responses rather than guaranteed real-world outcomes. Future studies should incorporate regionally stratified and consumption-weighted sampling, primary household measurements of ingredient sourcing, serving sizes, cooking duration, and energy use, updated region-specific emergy coefficients, probabilistic uncertainty analyses, and household pilot studies evaluating the economic, sensory, and cultural feasibility of the proposed optimization measures.

## 5. Conclusions

This study developed an emergy-based assessment framework encompassing agricultural production, ingredient processing, and household cooking to evaluate the sustainability of 341 CHCDs and examine their potential optimization pathways. The mean emergy input was 5.93 × 10^12^ sej·dish^−1^, with nonrenewable purchased inputs accounting for 77.99% of the total. Ingredients, oil and sauces, and household cooking accounted for 69.17%, 21.69%, and 9.14% of the total emergy input, respectively. The mean values of the emergy sustainability index (ESI), transformity (TRA), emergy consumption intensity (ECI), and food processing intensity (FPI) were 0.26, 1.02 × 10^6^ sej·J^−1^, 1.45 × 10^12^ sej·serving^−1^, and 4.64 × 10^11^ sej·serving^−1^, respectively. Three main patterns emerged from the analysis. First, longer ingredient production chains were associated with greater upstream emergy accumulation. Animal-derived ingredients were particularly emergy-intensive, reflecting multiple trophic transformations and the strong reliance of animal production on feed. Second, oil, sauces, and cooking energy constituted major consumption-side emergy hotspots, partially offsetting the lower upstream resource requirements of plant-based dishes and increasing their overall processing intensity. Third, regional differences in resource endowments, culinary traditions, and dietary practices were associated with pronounced spatial heterogeneity in sustainability performance.

Five standalone interventions—integrated crop–livestock recycling (ICLR), enhanced mechanization and energy efficiency (EMEE), technological advancement and appropriate scaling-up (TAAS), moderate oil and sauce reduction (MOSR), and enhanced cooking efficiency (ECE)—produced distinct changes in the key emergy indices. Their integration into the combined optimization scenario (COS) produced the most comprehensive improvements across the four indicators. Under COS, ESI increased to 0.31, representing a 19.34% improvement; the increase in ESI slightly exceeded the summed improvements achieved by the standalone interventions (18.81%), indicating a modest superadditive effect. Accordingly, a transition toward more sustainable CHCDs can be pursued through a dual strategy that combines coordinated optimization throughout the value chain with interventions tailored to specific dish categories and regional cuisines. At the system level, improvements in the renewability and resource-use efficiency of upstream production should be pursued, alongside optimization of resource inputs and cooking processes at the consumption stage. At the dish-category level, intervention priorities should reflect the principal limitations in the emergy input structures of different dish categories. Ingredient selection should explicitly account for the associated resource consumption and environmental pressures. From an emergy perspective, a greater inclusion of mixed plant–animal dishes in place of some animal-based dishes may represent a potential option for improving resource-use efficiency. Overall, dish-level emergy accounting and optimization enable a more comprehensive assessment of the resource use and environmental pressures associated with dietary consumption and provide a quantitative basis for designing agri-food sustainability strategies that improve resource-use efficiency and system sustainability while accounting for Chinese culinary traditions.

## Figures and Tables

**Table 1:** Equations used to calculate the emergy of resource inputs.

Input Category	Emergy Calculation Formula
Natural inputs	Solar emergy = S × average solar radiation (J·m^−2^·yr^−1^) × (1 − albedo) × UEV
	Wind emergy = S × height (m) × air density (kg·m^−3^) × eddy diffusion coefficient (m^2^·s^−1^) × wind speed gradient (s^−1^) × UEV
	Rain emergy (chemical potential) = S × average rainfall (m·yr^−1^) × Gibbs free energy of rainwater (J·kg^−1^) × water density (kg·m^−3^) × UEV
	Rain emergy (geopotential) = S × average altitude (m) × average rainfall (m·yr^−1^) × water density (kg·m^−3^) × gravitational acceleration (m·s^−2^) × UEV
	Earth-cycle emergy = S × annual heat flow (J·m^−2^·yr^−1^) × UEV
	Net loss of topsoil emergy = S × erosion rate (kg·m^−2^·yr^−1^) × soil organic matter content (kg·kg^−1^) × organic matter energy (J·kg^−1^) × UEV
Purchased inputs	E_i_ = f_i_ × UEV_i_, where E_i_ denotes the emergy of the ith purchased input and f_i_ denotes the corresponding energy (J), mass (g), or monetary flows (USD).

Note: S denotes the system area (m^2^); UEV denotes the item-specific unit emergy value for each natural input; and UEV_i_ denotes the unit emergy value of the ith purchased input.

**Table 2:** Unit emergy values and renewability factors used in the emergy analysis of Chinese home-cooked dishes.

Category	Item	Unit	Unit Emergy Value (sej·unit^−1^)	Renewability Factor	References
Renewable natural inputs					
Solar		J	1.00	1.00	[10]
Rain (chemical potential)		J	2.31 × 10^4^	1.00	[10]
Rain (geopotential)		J	1.33 × 10^4^	1.00	[10]
Wind		J	8.00 × 10^2^	1.00	[30]
Earth cycle		J	4.90 × 10^3^	1.00	[30]
Nonrenewable natural inputs					
Agricultural water		J	1.92 × 10^5^	0.00	[31]
Net loss of topsoil		J	9.36 × 10^4^	0.00	[31]
Purchased inputs					
Piglets		$	3.17 × 10^12^	0.05	[32]
Calves		$	3.17 × 10^12^	0.05	[32]
Lambs		$	3.17 × 10^12^	0.05	[32]
Chicks		$	3.17 × 10^12^	0.05	[32]
Fixed assets		$	3.17 × 10^12^	0.05	[32]
Veterinary biosecurity		$	3.17 × 10^12^	0.05	[32]
Concentrate feed		g	1.92 × 10^9^	0.17	[33]
Roughage		J	9.40 × 10^4^	0.11	[34]
Nitrogen fertilizer		g	4.83 × 10^9^	0.00	[10]
Phosphate fertilizer		g	4.95 × 10^9^	0.00	[10]
Potash fertilizer		g	1.40 × 10^9^	0.00	[10]
Compound fertilizer		g	3.56 × 10^9^	0.00	[10]
Organic fertilizer		g	2.96 × 10^8^	0.80	[31]
Pesticide		g	2.47 × 10^10^	0.00	[35]
Electricity		J	1.13 × 10^5^	0.09	[36]
Coal		J	7.67 × 10^4^	0.00	[37]
Diesel		J	1.17 × 10^5^	0.00	[37]
Natural gas		J	1.35 × 10^5^	0.00	[37]
Labor		J	5.73 × 10^6^	0.60	[38]
Cooking oil		g	9.17 × 10^9^	0.36	[10,39]
Sauces		g	1.38 × 10^10^	0.19	[10,40,41]
Major output					
Chinese home-cooked dishes		J			

Note: The emergy baseline adopted in this study was 12 × 10^24^ sej·yr^−1^. Renewable natural emergy was determined in accordance with emergy algebra. Applicable independent first-order inputs, including solar radiation and Earth cycle, were first summed. This sum was then compared with the largest applicable higher-order co-product, and the larger non-overlapping contribution was retained to avoid double counting.

**Table 3:** Parameter adjustments for the five individual CHCD system optimization scenarios.

Scenarios	Parameter Adjustments	References
ICLR	The renewability factors of concentrate feed and electricity were increased to 0.251 and 0.213, respectively.	[44]
EMEE	Energy inputs were reduced by 30%.	[31]
TAAS	Fertilizer, pesticide, and feed inputs were reduced by 20%, 30%, and 10%, respectively.	[5,45]
MOSR	Cooking oil and sauce inputs were reduced by 10% and 20%, respectively.	[46,47,48]
ECE	Energy consumption during cooking was reduced by 28%.	[49,50]

## Data Availability

The original contributions presented in this study are included in the article. Further inquiries can be directed to the corresponding authors.

## Abbreviations

The following abbreviations are used in this manuscript:

CHCDs	Chinese home-cooked dishes
PBD	Plant-based dishes
ABD	Animal-based dishes
MPAD	Mixed plant–animal dishes
UEV	Unit emergy value
EYR	Emergy yield ratio
ELR	Environmental loading ratio
ESI	Emergy sustainability index
TRA	Transformity
ECI	Emergy consumption intensity
FPI	Food processing intensity
BAU	Business-as-usual
ICLR	Integrated crop–livestock recycling
EMEE	Enhanced mechanization and energy efficiency
TAAS	Technological advancement and appropriate scaling-up
MOSR	Moderate oil and sauce reduction
ECE	Enhanced cooking efficiency
COS	Combined optimization scenario

## Appendix A

**Table A1:** Twenty representative Chinese home-cooked dish recipes selected from the full dataset (*n* = 341).

Dish (Category)	Key Ingredients	Cooking Oil and Sauces	Cooking Methods	Cooking Time	Region of Origin	Number of Servings
Paocai (PBD)	Kidney beans: 0.12 kg; Cabbage: 0.227 kg; White radish: 0.25 kg; Radish: 0.122 kg; Pepper: 0.2 kg	Oil: 0 g; Sauce: 60 g	Boiling + marinating	High heat: 5 min	Sichuan	4
Di san xian (PBD)	Eggplant: 0.25 kg; Potato: 0.2 kg; Pepper: 0.1 kg	Oil: 60 g; Sauce: 45 g	Deep-frying + stir-frying	Medium heat: 7 min; High heat: 8 min	Northeast China	4
Peanuts in aged vinegar (PBD)	Peanut: 0.2 kg	Oil: 100 g; Sauce: 120 g	Deep-frying + mixing	Low heat: 5 min	Shanxi	6
Braised eggplant in soy sauce (PBD)	Eggplant: 0.5 kg	Oil: 80 g; Sauce: 35 g	Red braising	Low heat: 3 min; Medium heat: 5 min; High heat: 3 min	Nationwide	4
Sweet congee with glutinous rice and wheat grains (PBD)	Glutinous rice: 0.2 kg; Wheat grains: 0.15 kg	Oil: 0 g; Sauce: 30 g	Boiling	Low heat: 80 min; High heat: 10 min	Guangdong	4
Smashed cucumber (PBD)	Cucumber: 0.4 kg	Oil: 10 g; Sauce: 25 g	Mixing	0 min	Nationwide	4
Braised pork meatballs in brown sauce (ABD)	Lean pork: 0.3 kg; fat pork: 0.2 kg	Oil: 60 g; Sauce: 69.5 g	Deep-frying + braising	Low heat: 5 min; Medium heat: 7 min	Shanghai	6
Stir-fried pork kidney (ABD)	Pork kidney: 0.2 kg	Oil: 35 g; Sauce: 72.5 g	Pan-frying + stir-frying	High heat: 5 min	Shandong	4
Hand-grabbed mutton (ABD)	Mutton ribs: 1 kg	Oil: 30 g; Sauce: 60 g	Stewing	Low heat: 90 min; High heat: 5 min	Inner Mongolia	4
Daokou braised chicken (ABD)	Chicken: 1.2 kg	Oil: 100 g; Sauce: 162 g	Deep-frying + braising	Low heat: 60 min; High heat: 15 min	Henan	4
Beef brisket braised in red wine (ABD)	Beef brisket: 0.6 kg	Oil: 15 g; Sauce: 370 g	Pan-frying + braising	Low heat: 45 min; High heat: 20 min	Hong Kong	4
Dongpo pork (ABD)	Boneless pork belly with skin: 0.5 kg	Oil: 15 g; Sauce: 480 g	Braising + steaming	Low heat: 123 min; High heat: 37 min	Zhejiang	4
Crispy lamb leg (ABD)	Lamb leg: 1 kg	Oil: 100 g; Sauce: 65 g	Stewing + deep-frying	Medium heat: 10 min; High heat: 60 min	Xinjiang	4
Stir-fried tomatoes and eggs (MPAD)	Tomato: 0.4 kg; Egg: 0.15 kg	Oil: 35 g; Sauce: 20 g	Stir-frying	Medium heat: 2 min; High heat: 3 min	Nationwide	4
Stir-fried pork with peppers (MPAD)	Pepper: 0.25 kg; Pork tenderloin: 0.2 kg	Oil: 40 g; Sauce: 35 g	Stir-frying	Medium heat: 2 min; High heat: 4 min	Hunan	4
Crumbled flatbread in mutton soup (MPAD)	Boneless lamb leg: 0.6 kg; Flatbread: 0.02 kg	Oil: 0 g; Sauce: 12 g	Boiling + stewing	Low heat: 75 min; High heat: 15 min	Shaanxi	4
Mapo tofu (MPAD)	Tofu: 0.5 kg; Minced pork: 0.1 kg	Oil: 50 g; Sauce: 47.5 g	Pan-frying + stir-frying	Low heat: 2 min; Medium heat: 5 min	Sichuan	4
Minchi (MPAD)	Minced pork: 0.15 kg; Minced beef: 0.1 kg; Potato: 0.2 kg; Egg: 0.1 kg	Oil: 45 g; Sauce: 30 g	Stir-frying + braising	Medium heat: 10 min; High heat: 2 min	Macao	3
Steamed pork with seasoned rice flour (MPAD)	Pork shoulder with skin: 0.45 kg; White rice: 0.12 kg; Dried tofu skin: 0.04 kg	Oil: 9 g; Sauce: 135 g	Steaming	Low heat: 60 min; Medium heat: 40 min	Anhui	4
Zhajiang noodles (MPAD)	Lean pork: 0.2 kg; Noodles: 0.6 kg; Cucumber: 0.1 kg	Oil: 45 g; Sauce: 100 g	Stir-frying + boiling	High heat: 8 min	Beijing	4

**Table A2:** Provincial and regional origins of the 341 Chinese home-cooked dishes included in this study.

Region of Origin	Number of Dishes, *n*
Anhui	11
Beijing	12
Chongqing	2
Fujian	7
Gansu	2
Guangdong	49
Guangxi	2
Guizhou	2
Hainan	3
Hebei	3
Henan	6
Hong Kong	25
Hubei	6
Hunan	15
Inner Mongolia	2
Jiangsu	8
Jiangxi	11
Macao	1
Nationwide	62
Ningxia	2
Northeast	12
Qinghai	2
Shaanxi	7
Shandong	16
Shanghai	12
Shanxi	3
Sichuan	31
Taiwan	7
Tianjin	2
Tibet	2
Xinjiang	5
Yunnan	2
Zhejiang	9
Total	341

Note: “Nationwide” refers to dishes that are not attributed to a specific provincial-level administrative region. “Northeast” comprises the three provinces of Heilongjiang, Jilin, and Liaoning.

**Table A3:** Standardized mass equivalents for ingredients without explicit mass information in the original recipes.

Ingredient	Household Measure	Equivalent Mass (g)
Fried tofu puff	1 piece	12.5
Red fermented bean curd	1 cube	15
Tofu skin	1 piece	40
Dried tofu	1 piece	25
Five-spice dried tofu	1 piece	30
Chinese cabbage leaf	1 leaf	30
Potato	1 whole	213
Cucumber	1 whole	201
Carrot	1 whole	61
Fresh chili pepper	1 whole	50
Cabbage	1 head	908
Chinese cabbage	1 head	900
Dried chili pepper	1 piece	3
Peas	1 tablespoon	20
Eggplant	1 whole	250
White radish	1 whole	400
Tomato	1 whole	123
Apple	1 whole	109
Corn	1 ear	90
Boneless, skin-on pork belly	1 piece	500
Egg	1 whole	50
Pork tongue	1 whole	250
Pork heart	1 whole	250
Chinese cured sausage	1 link	50
Pork large intestine	1 section	100
Pork kidney	1 whole	100
Pork tail	1 whole	100
Lamb chop	1 small piece	50
Boneless, skinless chicken thigh	1 piece	100
Boneless, skinless chicken breast	1 piece	150
Pork rib	1 piece	100
Bone-in, skin-on chicken leg	1 leg	170
Chicken liver	1 piece	20
Chicken wing	1 wing	35
Egg white	from 1 egg	30
Egg yolk	from 1 egg	20
Pork marrow bone	1 piece	600
Pork stomach	1 whole	700
Cornstarch	1 tablespoon	8
Granulated sugar	1 tablespoon	8
Spring roll wrappers	8 sheets	120

**Table A4:** Standardized electricity and natural gas consumption rates and corresponding energy inputs at different heat settings during cooking.

Energy Source	Heat Setting	Consumption Rate	Energy Input (J·min^−1^)
Electricity	High	0.035 kWh·min^−1^	1.26 × 10^5^
Electricity	Medium	0.020 kWh·min^−1^	7.20 × 10^4^
Electricity	Low	0.00667 kWh·min^−1^	2.40 × 10^4^
Natural gas	High	0.45 m^3^·h^−1^	2.70 × 10^5^
Natural gas	Medium	0.25 m^3^·h^−1^	1.50 × 10^5^
Natural gas	Low	0.10 m^3^·h^−1^	6.00 × 10^4^

## Appendix B

According to the recommendations of the International Society for the Advancement of Emergy Research, the latest global emergy baseline established in 2016 (12 × 10^24^ sej·yr^−1^) was adopted as the reference baseline in this study. When a unit emergy value (UEV) was obtained from a source using a different global emergy baseline, it was rescaled according to the ratio between the baseline used in the original reference and the latest global emergy baseline. Specifically, UEVs reported on the basis of 9.26 × 10^24^ sej·yr^−1^, 9.44 × 10^24^ sej·yr^−1^, and 15.83 × 10^24^ sej·yr^−1^ were multiplied by factors of 1.30, 1.27, and 0.76, respectively, to convert them to the common baseline of 12 × 10^24^ sej·yr^−1^.

For UEVs expressed on an energy basis (sej·J^−1^) or a mass basis (sej·g^−1^), priority was given to estimates reported by Odum, Brown and Ulgiati, and their research groups, because these groups have conducted extensive foundational work in emergy accounting and most subsequent studies have adopted or built upon their UEV estimates, particularly for natural resources and fossil energy. For agricultural inputs, authoritative studies conducted at the Chinese national scale were preferentially selected, including those reporting UEVs for pesticides, concentrate feed, and organic fertilizers. Where national-scale Chinese estimates were unavailable, values from regional studies conducted in China were used. For monetary inputs expressed in USD, the most recent emergy-to-money ratio (EMR) available for China was adopted. Monetary values were adjusted for inflation using the GDP deflator, with an adjustment factor of 1.47 applied in this study.

### Appendix B.1. Calculation of UEVs for Edible Oils

Because directly reported UEVs for refined peanut, soybean, and corn oils were unavailable or insufficiently documented for consistent use, their UEVs were estimated by accounting for the emergy embodied in the corresponding oil-bearing raw materials and the emergy associated with energy consumed during oil processing (Table A5). The calculation was performed on the basis of 1 t (1000 kg) of edible oil.

The simplified calculation included two major components: (1) the emergy embodied in the oil-bearing raw material and (2) the emergy associated with energy consumption during oil processing. Accordingly, the emergy included in the calculation was estimated as:

(A1) $$Em_{oil,i}=Em_{raw,i}+Em_{proc,i}$$

where $Em_{oil,i}$ is the estimated emergy input associated with the production of 1 t of edible oil *i* (sej), $Em_{raw,i}$ is the emergy embodied in the corresponding oil-bearing raw material (sej), and $Em_{proc,i}$ is the emergy associated with processing energy consumption (sej).

#### Appendix B.1.1. Emergy of Oil-Bearing Raw Materials

For each type of edible oil, the mass of raw material required to produce 1 t of refined oil was estimated from the corresponding oil yield:

(A2) $$M_{\mathrm{raw},i}=\frac{1000}{Y_{i}}$$

where $M_{\mathrm{raw},i}$ is the required mass of raw material (kg) and $Y_{i}$ is the mass of refined oil obtained per unit mass of raw material, expressed as a decimal fraction.

The emergy embodied in the raw material was subsequently calculated as:

(A3) $$Em_{raw,i}=M_{raw,i}\times Q_{raw,i}\times UEV_{raw,i}$$

where $Q_{raw,i}$ is the calorific value of the oil-bearing raw material (J·kg^−1^), and $UEV_{raw,i}$ is its unit emergy value (sej·J^−1^).

#### Appendix B.1.2. Emergy Associated with Oil Processing

Processing energy intensity was expressed as kilograms of standard coal equivalent per tonne of refined oil (kgce·t^−1^ of oil). The processing energy intensities adopted for peanut, soybean, and corn oils were 380,320, and 420 kgce·t^−1^ of oil, respectively. Because the calculations were based on 1 t of refined oil, the corresponding quantities of standard coal equivalent used in the calculation were 380,320, and 420 kgce, respectively.

With an energy conversion factor of 29.3 × 10^6^ J·kgce^−1^, the processing energy input for 1 t of edible oil was calculated as:

(A4) $$E_{\mathrm{proc},i}=W_{i}\times 29.3\times 10^{6}$$

where $W_{i}$ is the quantity of standard coal equivalent required to process 1 t of edible oil *i* (kgce) and $E_{\mathrm{proc},i}$ is the corresponding processing energy input (J).

The emergy associated with processing energy consumption was subsequently calculated as:

(A5) $$Em_{\mathrm{proc},i}=E_{\mathrm{proc},i}\times UEV_{\mathrm{proc}}$$

where $UEV_{\mathrm{proc}}$ is the common UEV adopted for processing energy, with a value of 8.5 × 10^4^ sej·J^−1^.

The adopted processing energy intensities were assumed to encompass pressing and refining for peanut oil; solvent extraction and refining for soybean oil; and germ separation, solvent extraction, and refining for corn oil.

#### Appendix B.1.3. Derivation of Edible Oil UEVs

A common calorific value of 37.0 × 10^6^ J·kg^−1^ was adopted for the three edible oils. Therefore, the energy content of 1 t of edible oil was calculated as:

(A6) $$E_{\mathrm{oil}}=1000\times 37.0\times 10^{6}=3.7\times 10^{10}\;J$$

The energy-based UEV of each edible oil was subsequently calculated by dividing the estimated emergy input by the energy content of the final oil product:

(A7) $$UEV_{\mathrm{oil},i}=\frac{Em_{\mathrm{raw},i}+Em_{\mathrm{proc},i}}{E_{\mathrm{oil}}}$$

where $UEV_{\mathrm{oil},i}$ is expressed in sej·J^−1^. For use in dish-level emergy accounting, the energy-based UEV was further converted to a mass-based value:

(A8) $$UEV_{oil,i}^{m}=UEV_{oil,i}\times 3.70\times 10^{4}$$

where 3.70 × 10^4^ J·g^−1^ is the calorific value of edible oil expressed on a mass basis, and $UEV_{oil,i}^{m}$ is the corresponding mass-based UEV, expressed in sej·g^−1^.

**Table A5:** Parameters used to estimate the UEVs of edible oils.

Parameter	Peanut Oil	Soybean Oil	Corn Oil
Oil-bearing raw material	Peanut	Soybean	Corn
Calorific value of raw material (J·kg^−1^)	23.8 × 10^6^	17.5 × 10^6^	16.3 × 10^6^
Original UEV (sej·J^−1^)	6.90 × 10^4^	6.90 × 10^4^	1.81 × 10^4^
Rescaled UEV (sej·J^−1^)	8.94 × 10^4^	8.94 × 10^4^	2.35 × 10^4^
Oil yield, Y (%)	42	18	3.5
Raw material required (kg·t^−1^ of oil)	2380.95	5555.56	28,571.43
Processing energy (kgce·t^−1^ of oil)	380	320	420
Major processing stages	Pressing and refining	Solvent extraction and refining	Germ separation, solvent extraction, and refining

### Appendix B.2. Calculation of UEVs for Sauces

The UEVs for sauces were estimated using the same accounting approach as that applied to edible oils, with emergy inputs from both raw-material production and food processing considered. Given the substantial differences in raw-material composition and processing techniques among condiments such as fermented broad-bean paste and sweet sauces, oil-crop and sugar-crop pathways were selected to approximate the range of raw-material inputs. Their emergy contributions were subsequently combined with representative energy consumption during sauce processing.

At the raw-material stage, the mean energy-based UEV of the representative oil crops, after rescaling to the global emergy baseline adopted in this study, was approximately 1.06 × 10^5^ sej·J^−1^. A mean dry-matter calorific value of approximately 22.5 kJ·g^−1^ and a mean recoverable oil fraction of approximately 18% were used as representative conversion parameters for the oil-crop pathway. For the sugar-crop pathway, the mean mass-based UEV of the representative sugar crops, after rescaling to the same global emergy baseline, was approximately 5.0 × 10^8^ sej·g^−1^, and a mean recoverable sugar fraction of approximately 12% was adopted. The raw-material emergy contributions of the two representative pathways were calculated using the same yield-based procedure applied to edible oils in Equations (A2) and (A3). These pathways were used as proxies for the range of sauce raw-material inputs rather than as representations of the precise formulation of any individual sauce.

At the processing stage, representative energy requirements for sugar processing and sweet-sauce processing were used to characterize the range of processing intensity. These energy requirements were approximately 6.90 and 4.20 MJ·kg^−1^, respectively, with their mean used to represent typical processing conditions. The corresponding processing emergy was calculated using the same processing-energy conversion procedure and processing-energy UEV described in Equations (A4) and (A5). The raw-material and processing emergy inputs were then summed, and the resulting emergy was normalized by the mass of the final sauce product to obtain a mass-based sauce UEV, following the same general accounting framework used for edible oils.
